# Microsatellite and Mitochondrial DNA Reveal Spatial Genetic Variation and Domestic Cat Admixture in Hungarian European Wildcats

**DOI:** 10.3390/ani16203166

**Published:** 2026-10-09

**Authors:** Péter Fehér, Krisztián Frank, Nóra Ninausz, Fanni Hámor, Lilla Sándorová, Péter Gombkötő, Virág Lilla Burányi, Zsolt Biró, Szilvia Kusza, Zoltán Bagi, Péter Szabó, Borbála Major, Zsuzsanna Szőke, Endre Barta, Viktor Stéger

**Affiliations:** 1 Department of Genetics and Genomics, Institute of Genetics and Biotechnology, Hungarian University of Agriculture and Life Sciences, H-2100 Gödöllő, Hungary; feher.peter.arpad@uni-mate.hu (P.F.); sandorova.lilla@uni-mate.hu (L.S.); 2 Department of Health and Nursing Sciences, Archiepiscopal College of Veszprém, H-8200 Veszprém, Hungary; krisz.frank.biol@gmail.com; 3 Doctoral School of Agricultural and Food Sciences, Hungarian University of Agriculture and Life Sciences, H-2100 Gödöllő, Hungary; nora.ninausz@gmail.com (N.N.); buranyi.virag@bkvp.hu (V.L.B.); 4 mcule.com Ltd., H-1115 Budapest, Hungary; hamor.fanni@gmail.com; 5 Bükk National Park Directorate, H-3304 Eger, Hungary; gombkoto.peter@bnpi.hu; 6 Budakeszi Wildlife Park Non-Profit Ltd., H-2092 Budakeszi, Hungary; szabo.peter@bkvp.hu; 7 Department of Wildlife Biology and Management, Institute for Wildlife Management and Nature Conservation, Hungarian University of Agriculture and Life Sciences, H-2100 Gödöllő, Hungary; biro.zsolt@uni-mate.hu; 8 Centre for Agricultural Genomics and Biotechnology, Faculty of Agricultural and Food Sciences and Environmental Management, University of Debrecen, H-4032 Debrecen, Hungary; kusza@agr.unideb.hu (S.K.); bagiz@agr.unideb.hu (Z.B.); 9 Duna-Ipoly National Park Directorate, H-1121 Budapest, Hungary; majorb@dinpi.hu; 10 Department of Animal Biotechnology, Institute of Genetics and Biotechnology, Hungarian University of Agriculture and Life Sciences, H-2100 Gödöllő, Hungary; ferenczine.szoke.zsuzsanna@uni-mate.hu; 11 Department of Biochemistry and Molecular Biology, Faculty of Medicine, University of Debrecen, H-4032 Debrecen, Hungary

**Keywords:** conservation genetics, *Felis silvestris*, Bayesian clustering, spatial genetic structure, *ND5*, Hungary

## Abstract

The European wildcat (*Felis silvestris*) is threatened due to habitat loss and hybridisation with domestic cats (*Felis catus*), representing a significant conservation challenge in Hungary. We genotyped 118 field-collected cats from seven Hungarian sub-regions and 30 domestic cat references at 14 autosomal microsatellites, and sequenced a 669 bp mitochondrial *ND5* fragment from the field samples. At K = 2, STRUCTURE assigned 84 field samples to the wildcat-associated cluster, 18 to the domestic-cat-associated cluster, and 16 (13.6%) to an intermediate membership interval. The combined non-wildcat proportion (28.8%) is not a hybridisation rate. Intermediate assignments fell to 10 at K = 3, demonstrating model dependence. Simulations recovered parental, F1 and F2 genotypes, but most second-generation and all third-generation backcrosses were assigned to a parental cluster. Genetic and geographic distances were significantly but weakly associated (Mantel *r* = 0.118, *p* = 0.005). Eight *ND5* haplotypes were identified; dw4 predominated in the North Hungarian Mountains and w1 in Southern Transdanubia. These maternal patterns are compatible with historical lineage retention and/or introgression, but a single locus cannot date or distinguish these processes. The results support targeted genetic monitoring while highlighting the need for ancestry-informative SNP or genome-wide validation before management decisions are based on individual hybrid classifications.

## Simple Summary

The European wildcat is threatened by the loss of suitable forest habitats and hybridisation with domestic cats in Hungary. This is a severe issue because wildcats play an important ecological role in preventing the overpopulation of small mammals. Our research aimed to assess the genetic status, spatial distribution, and extent of domestic cat influence within the Hungarian population. We examined 118 individuals from various regions of the country. Our results revealed that while many individuals belong to the wildcat group, notable proportion are free-ranging domestic cats or show mixed ancestry. Different maternal lineages dominate in different regions, reflecting a complex history, although basic genetic tools cannot pinpoint the exact timing of these past interactions. Our study confirms that while conservation monitoring is essential, managers need more advanced genetic tools before making critical decisions about individual classification. This is highly valuable to society, as protecting wildcats is fundamental to maintaining the long-term, healthy balance of forest ecosystems.

## 1. Introduction

In the current unprecedented extinction crisis, carnivores, and felids in particular, are exposed to the highest extinction risk among mammals [1]. Historically, the European wildcat has been subjected to severe hunting and trapping. This factor, combined with habitat loss, has resulted in population declines throughout the species’ entire distribution [2,3,4]. The primary threats include the loss of potential habitats [5,6], human-induced mortality—particularly roadkills [7,8,9] and poaching [9]—as well as the loss of allelic variants due to genetic drift [3]. A major issue is hybridisation with the domestic cat [4,7,10,11,12,13,14,15,16,17,18], which is hypothesized to have already led to the local extinction of certain populations. Furthermore, free-ranging domestic cats can affect wildcats indirectly through predation on shared small-mammal prey [14,15,19], exploitative and interference competition [20], disturbance, and the transmission of various diseases [21]. As a key mammalian mesocarnivore, the wildcat plays a crucial role in ecosystem functioning, particularly in regulating small mammal populations through top-down control in the absence of apex predators [22]. However, the increasing population of free-ranging and feral domestic cats exerts severe predation pressure on native wildlife globally [23]. Furthermore, hybridisation can fundamentally alter this essential ecological function, as evidenced by significant trophic niche overlaps and dietary competition between wildcats, feral cats, and their hybrids in Hungary [14].

Habitat loss and fragmentation over the past decades have led to a decrease in the genetic diversity of populations [4]. Genetic studies have revealed that gene flow from domestic cats to wildcats varies in intensity across Europe, exhibiting significant local differences that can likely be attributed to historical or ecological factors [17,24]. For instance, low levels of hybridisation have been found in Italian, Bulgarian, Portuguese, and German populations [11,25,26,27], whereas high hybridisation rates have been detected in the wildcat populations of Scotland and Hungary [4,24,28]. Examining the European wildcat population, five distinct biogeographical groups have been identified: the Northern Balkans, Italy and Sicily, Central Germany, Central Europe, and the Iberian Peninsula [26,29].

Information regarding the distribution of the wildcat in Hungary is limited. The population can be geographically divided into three main areas: the most stable of these spans from the Bakony Mountains to the Zemplén Mountains (connecting to the Northern Carpathians); the Transtisza region and the floodplain forests of the Tisza River (connecting to the Eastern Carpathians); and in the south, the Mecsek region connected to the Danube floodplain. In these regions, human land use and intensive agricultural landscapes pose a dual threat. On the one hand, a dense road network and human infrastructure heavily fragment suitable forest habitats, acting as barriers that hinder natural gene flow between these core wildcat populations [5,6]. On the other hand, the same landscape structure produces an extensive network of forest edges bordering settlements and farmland; these edges provide routes along which domestic cats can penetrate into previously isolated wildcat habitats, thereby increasing the opportunity for contact and hybridisation [6]. Investigating the hybridisation patterns of the Hungarian population, ref. [4] concluded that the samples from this region comprise a combination of variable phenotypes and genotypes, which may be the result of long-term hybridisation and introgression. Based on a study utilizing microsatellite loci by [30], hybridisation was detectable in 8% of the individuals; however, using linked loci, this value was higher, clearly confirming the ongoing presence of hybridisation in the Hungarian population. This extensive introgression presents a significant challenge for practical wildlife management. While the active regulation of feral cat populations is legally and ecologically essential for wildcat conservation in Hungary, traditional morphological identification (phenotype) is no longer reliable for distinguishing pure wildcats from hybrids in the field due to advanced introgression [31,32]. Consequently, conservation managers are increasingly dependent on robust genetic tools to precisely identify admixed individuals and to inform targeted conservation and removal efforts [33].

Biogeographic and phylogeographic patterns are key factors in understanding the evolutionary histories of taxa and in identifying appropriate conservation management units for sustainable preservation [16,34]. The contemporary genetic structure of the European wildcat is a direct consequence of the climatic oscillations of the Pleistocene epoch. These periods of isolation promoted genetic drift and the fixation of unique mitochondrial haplotypes [35]. To date, only a few molecular studies have investigated the phylogenetic and phylogeographic patterns of *Felis silvestris* in Europe. These investigations mainly used informative mitochondrial regions (such as the Control Region or the NADH dehydrogenase subunits) to distinguish domestic cat and European wildcat lineages, and detected the rough presence of a continental and a Mediterranean clade within the latter [10,27,36,37,38,39]. Extensive analysis of mitochondrial markers, particularly the *ND5* gene, has reconstructed an exhaustive biogeographic history of the species across its entire distribution range. Five main genetic clusters have been identified, corresponding to distinct biogeographic groups [29]. The diversification of these lineages (referred to as Lineage W) began approximately 64,200 years ago, coinciding with the cooling phases of the late Pleistocene [35]. The isolated Scottish population, the Iberian cluster, and the South-Eastern European group represent the earliest branches of this diversification [35]. The Balkan Peninsula has served as a critical migration corridor between Asia Minor and Europe, acting as a refugium during the Last Glacial Maximum (LGM) [40]. However, contemporary Balkan wildcat populations exhibit surprisingly low mitochondrial diversity [35]. A contrasting case is found in the Sardinian wildcat (*F. s. lybica*). While located in Europe, genetic and archeological evidence traces the origins of these cats back to human-mediated dispersal of Near Eastern wildcats over 2000 years ago [41].

The identification of “pure” European wildcats versus domestic hybrids relies on a set of vetted mitochondrial polymorphisms. These markers are prioritized in forensic and conservation genetics because of their ability to detect maternal introgression even after multiple generations of backcrossing with wild males [42]. The *ND5* gene is arguably the most diagnostic portion of the mitogenome for distinguishing between wild and domestic lineages. Phylogenetic analyses have identified two primary lineages: Lineage W (Wild) and Lineage D (Domestic/Lybica) [35].

The aim of this study was to characterize the current genetic status of the Hungarian European wildcat population and to quantify the extent of domestic cat associated ancestry within it. To this end, we assessed genetic diversity, spatial variation, and domestic cat associated ancestry in field collected Hungarian cats using 14 autosomal microsatellites and a 669 bp fragment of mitochondrial *ND5*. We tested whether (i) field samples were differentiated from domestic cat references and showed spatially structured nuclear variation; (ii) intermediate STRUCTURE assignments varied among sub-regions; (iii) the marker panel could distinguish parental, F1, F2, and backcross genotypes in simulations; and (iv) mitochondrial haplotypes exhibited geographic heterogeneity. We expected early-generation hybrids to be more readily detected than advanced backcrosses and treated historical interpretations of mitochondrial lineages as hypotheses rather than direct temporal inferences.

## 2. Materials and Methods

### 2.1. Samples

**Figure 1:**
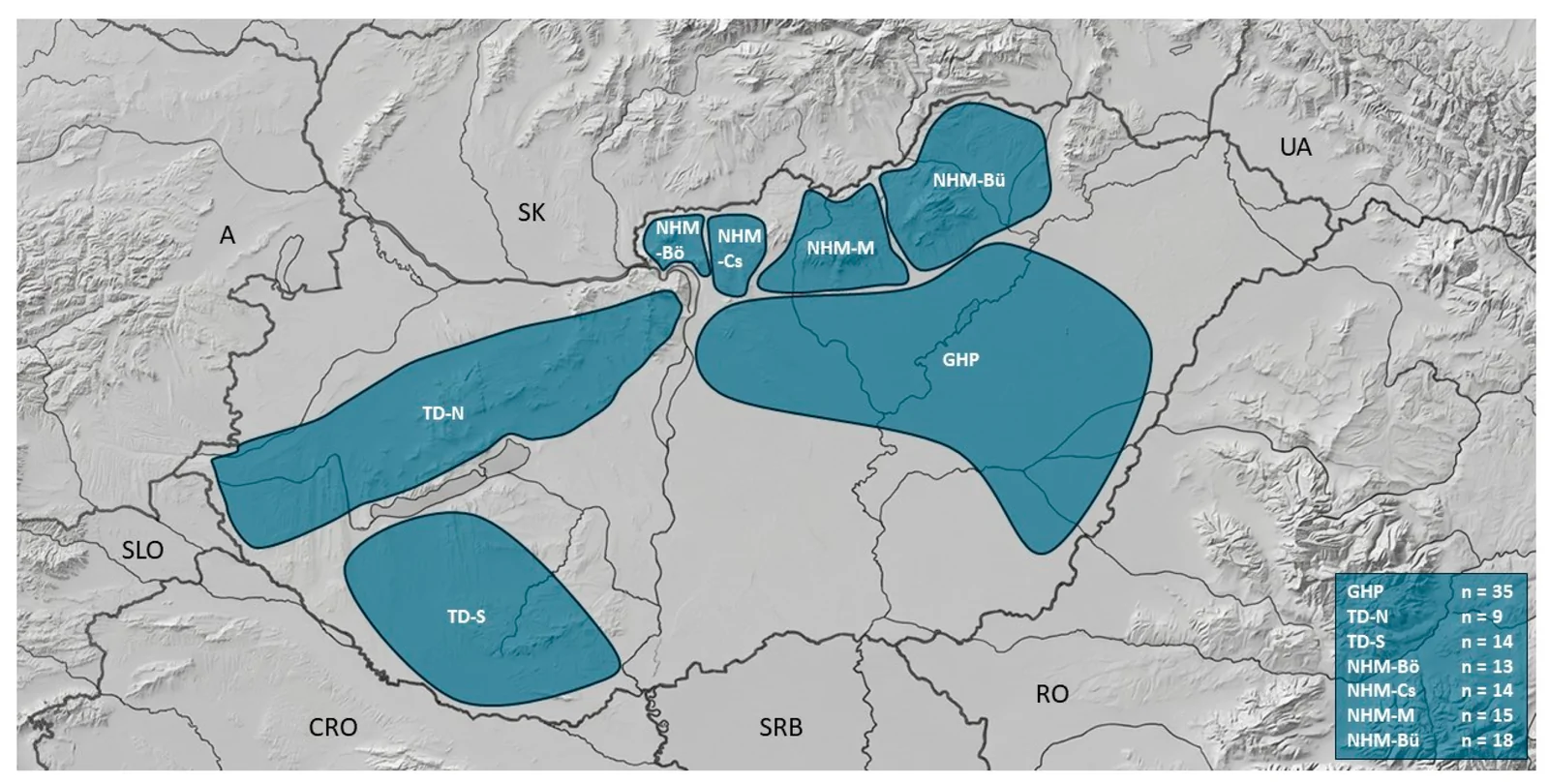
Sampling sites of wildcats in Hungary. GHP—Great Hungarian Plain, TD-S—Southern Transdanubia, TD-N—Northern Transdanubia, NHM-Bö—North Hungarian Mountains Börzsöny, NHM-Cs—North Hungarian Mountains Cserhát, NHM-M—North Hungarian Mountains Mátra, NHM-Bü—North Hungarian Mountains Bükk. A—Austria, SK—Slovakia, UA—Ukraine, RO—Romania, SRB—Serbia, CRO—Croatia, SLO—Slovenia.

Hair samples (*n* = 118) were collected between 2016 and 2026 from putative wildcats, predominantly from road-killed individuals. Reference domestic cat hair samples (*n* = 30) were obtained from live, owned domestic cats treated at veterinary clinics in and around Budapest; a small number of hairs with follicles was taken non-invasively during routine veterinary care or handling, with the prior informed consent of the owners (see Informed Consent Statement). Prior to analysis, all samples were stored in plastic bags at −20 °C. The field-collected samples were assigned to geographic groups based on the major geographical regions of Hungary and the core areas of the species’ national distribution range. Based on their geographic locations (Supplementary Table S1), the field-collected samples were categorized into the following groups: Great Hungarian Plain (GHP, *n* = 35), Northern Transdanubia (TD-N, *n* = 9), Southern Transdanubia (TD-S, *n* = 14), as well as the areas of the North Hungarian Mountains, Börzsöny (NHM-Bö, *n* = 13), Cserhát (NHM-Cs, *n* = 14), Mátra (NHM-M, *n* = 15), and Bükk (NHM-Bü, *n* = 18) Mountains (Figure 1).

### 2.2. Laboratory Procedures

DNA from samples was extracted using the MagCore HF16 Automated Nucleic Extractor with the MagCore Genomic DNA Tissue Kit (RBC Bioscience Corp., Taipei, Taiwan), according to the manufacturer’s protocol. The genomic DNA samples were stored at −20 °C.

For microsatellite analysis we amplified 14 loci (FCA043, FCA023, FCA097, FCA132, FCA096, FCA223, FCA698, FCA126, FCA229, FCA220, FCA090, FCA008, FCA045, F115) adapted from [43] (Supplementary Table S2), using multiplex PCR panels with the Qiagen Multiplex PCR Kit (QIAGEN GmbH, Hilden, Germany). The reaction mixture volume was 25 μL, containing Multiplex MasterMix (QIAGEN GmbH, Hilden, Germany), 60 ng of DNA, optimized amounts of primers at 10 μM concentration, and nuclease-free water. PCR was conducted in a LifeECO instrument (Bioer Technology Co., Ltd., Hangzhou, China). The PCR profile for all multiplexes was the following: 95 °C for 15 min; 30 cycles of 94 °C for 30 s, 58 °C for 30 s, 72 °C for 60 s, and final extension at 60 °C for 30 min. Fragment capillary analysis was conducted on Applied Biosystems 3500xL Genetic Analyzer instruments with LIZ500 size standard (Applied Biosystems, Foster City, CA, USA). The lengths of microsatellite fragments were described using Geneious Prime 2024.0.7 (Biomatters Ltd., Auckland, New Zealand) with Two Surrounding Peaks sizing method.

From the field samples, the mitochondrial NADH dehydrogenase subunit 5 gene (*ND5*; nucleotides 13,149–13,983 in the domestic cat mitochondrial genome; NCBI reference sequence NC_001700) was amplified and sequenced following the protocol of [35]. This mtDNA region is particularly suitable for phylogenetic reconstructions because it is highly discriminatory, containing seven diagnostic mutations that are useful for distinguishing between the European wildcat (*F. silvestris*) and domestic cat/African cat (*F. catus/lybica*) lineages, and furthermore, it does not contain nuclear mitochondrial segments (numts) and has low homoplasy [10,36]. Analysis of *ND5* was done using primers described by [42] (Forward: 5′-TGC CGC CCT ACA AGC AAT-3′, Reverse: 5′-TAA GAG ACG TTT AAT GGA GTT GAT-3′). The reaction mixture volume was 20 μL, it contained 3 μL of DNA diluted to the concentration of 15 ng/μL, 10 μL of Phire Animal Tissue PCR Buffer, 0.4 μL of Phire HS II DNA polymerase enzyme, 0.5 μL of each primer diluted to 10 μM and 5.6 μL nuclease free water. PCR was conducted in a LifeECO instrument (Bioer Technology Co., Ltd., Hangzhou, China). The PCR profile was the following: 98 °C for 5 min, 35 cycles of 98 °C for 10 s, 60.8 °C for 25 s, 72 °C for 25 s, and final extension at 72 °C for 5 min.

### 2.3. Data Analysis

#### 2.3.1. Microsatellite Analysis

Data analysis for microsatellites was used to analyze electropherograms and score allele sizes. The program MICRO-CHECKER ver. 2.2.3 [44] was used to assess null alleles, scoring errors and large allele dropout. To control for artefacts associated with the presence of null alleles, the Brookfield method [45] as implemented in the MICRO-CHECKER 2.2.3 [44] software was used. Moreover, estimates of *F*_ST_ confidence intervals considering and without considering null alleles were estimated and compared using the program FreeNA v1.0 [46]. Pairwise *F*_ST_ values for the eight populations were calculated using the G-statistics feature in GenAlEx v6.51b2 [47] with 9999 permutations. Since the ENA-corrected *F*_ST_ values for null alleles computed by FreeNA were of the same magnitude as the raw values, the statistical significance (*p*-values) between population pairs was determined based on an exact test performed on the raw dataset in GenAlEx with 9999 permutations. We used GenAlEx ver. 6.5 [47] to estimate allele frequency by locus and population, the number of alleles (Na), the number of effective alleles (Ne), observed and expected heterozygosity values (H_O_ and H_E_, respectively).

To assess potential admixture between different wildcat populations in Hungary, as well as between wildcat populations and domestic cats, we used the Bayesian clustering method implemented in version 2.3.4 of STRUCTURE [48] to determine the most likely number of genetic clusters, without predefining the populations. The Bayesian clustering method and Markov Chain Monte Carlo (MCMC) simulation were run using an admixture model and correlated allele frequencies. STRUCTURE was run with simulations of 10 runs for each value of K, with a burn-in period of 250,000 iterations and 750,000 replications for a number of genetic clusters (K) from one to seven.

The optimal number of clusters (K) was determined using the StructureSelector software (https://lmme.ac.cn/StructureSelector/, accessed on 6 October 2026) [49]. This involved calculating the second-order rate of change in the log probability of data (ΔK), as well as evaluating the four K-estimator statistics applying threshold values between 0.5 and 0.8 [50]. Following the determination of the most likely number of genetic clusters (K) using StructureSelector, the results of the 10 independent replicate runs for each K value were aligned and averaged using the CLUMPAK pipeline (https://lmme.ac.cn/StructureSelector/, accessed on 6 October 2026) [51] integrated within the StructureSelector platform. We applied the LargeKGreedy (https://lmme.ac.cn/StructureSelector/, accessed on 6 October 2026) algorithm with 2000 random input orders to compute the consensus individual membership proportions q^(i)^. These consensus q^(i)^ values were subsequently used to generate the final bar plots. For the K = 2 solution, field samples were classified as wildcat-associated when the membership coefficient to the wildcat cluster was q^(i)^ ≥ 0.75, as domestic cat-associated when q^(i)^ ≤ 0.25, and as putatively admixed for intermediate values (0.25 < q^(i)^ < 0.75). This interval is centred on the value of q^(i)^ = 0.5 expected for an F1 genotype and corresponds to the range conventionally applied in wildcat admixture studies [27].

To investigate the spatial genetic variability and patterns of the Hungarian wildcat population, a spatial Principal Component Analysis (sPCA) was performed [52]. For this analysis, individuals identified as domestic cats (q^(i)^ ≥ 0.75 assignment to the domestic cat cluster) based on the STRUCTURE results were excluded from the free-living samples, leaving only putatively admixed and wildcat individuals. The analysis was carried out in the R software 4.5.0 (R Development Core Team, 2020) environment using the adegenet (version 2.1.11) and spdep (version 1.4.2) packages [52], based on data from 14 microsatellite loci. Unlike traditional Principal Component Analysis (PCA), sPCA is a multivariate method that explicitly incorporates the geographic coordinates of individuals alongside their genetic profiles. To calculate spatial autocorrelation (Moran’s I), a distance-based connection network was defined. To ensure a continuous network where each sampling point has at least one neighbour, the threshold distance was determined based on the maximum distance between nearest neighbours. During the construction of the connection network, the minimum Euclidean distance was set to 0. The maximum threshold distance was calculated dynamically, based on the maximum of the nearest-neighbour distances between individuals (k = 1 neighbour criterion), thereby ensuring spatial continuity and that every examined individual had at least one neighbour during the analysis. The sPCA identified synthetic components that maximize the product of genetic variance and spatial autocorrelation, summarizing the spatial genetic structure of the population. For the visualization of global patterns, the values of the first global principal components were represented in geographic scale along a continuous colour scale. To statistically test for Isolation by Distance (IBD), a Mantel test [53] was performed with 999 permutations using the ade4package [54] in the R 4.5.0 statistical environment. This test examined the correlation between the genetic distance matrix (Euclidean distance) and the geographic distance matrix to determine the strength (r) and statistical significance (*p*) of the relationship.

To evaluate the statistical power of the STR marker set for detecting hybridisation between domestic and wildcats, we simulated parental and putatively admixed genotypes using recom-sim.py [55]. As baseline data for these simulations, we selected complete empirical microsatellite profiles (i.e., featuring no missing loci) that were assigned to either parental group (wildcat-associated or domestic-cat-associated) with a membership proportion q(i) ≥ 0.75 in the initial STRUCTURE analysis. For the K = 2 solution, field samples were classified as wildcat-associated when the consensus membership coefficient to the wildcat cluster was q(i) ≥ 0.75, as domestic-cat-associated when q(i) ≤ 0.25, and as putatively admixed for intermediate values (0.25 < q(i) < 0.75). This interval is centred on the value of q(i) = 0.5 expected for an F1 genotype, and the 0.75 threshold follows established practice in European wildcat admixture studies [27]. From the free-living samples, 84 individuals were assigned to the wildcat cluster based on this assignment probability; these, along with 30 domestic cats, were used as reference populations to generate the various genotype classes. We simulated a total of 400 genotypes across the following categories: two parental classes, F1 (parental × parental), F2 (F1 × F1), first-generation backcrosses (offspring of a first-generation hybrid × parental) and second-generation backcrosses (offspring of a first-generation backcross × parental) to both parental lineages (50 simulated genotypes per class). A subsequent STRUCTURE analysis was then performed on these simulated parental and putatively admixed genotypes. Consistent with the previous run, we applied the admixture model with correlated allele frequencies, utilizing a burn-in period of 250,000 iterations followed by 750,000 Markov Chain Monte Carlo (MCMC) iterations. In this step, however, we restricted the tested number of genetic clusters to K = 1 and K = 2, performing 10 independent runs for each K value. Within each K value, the most likely of the 10 independent replicates was chosen based on the Estimated Ln Prob of Data, with the highest value representing the optimal run.

#### 2.3.2. Mitochondrial Analysis

The *ND5* sequences of the 118 field samples were checked and corrected in BioEdit version 7.7.1.0 [56]. Nucleotide sequences were aligned by ClustalW implemented in MEGA12 (version 12.0.14) [57], and manually adjusted if needed. The alignment was trimmed to 669 bp in length (positions 13,243-13,911) to be comparable with the haplotypes identified by [35], as described in their Supplementary Table S1. The number of distinct haplotypes, haplotype and nucleotide diversities, and the number of polymorphic sites were computed with DnaSP version 6.12.03 [58]. Neutrality statistics (Tajima’s D and Fu and Li’s F*) were calculated using the pegas (version 1.4) package [59] in the R environment (version 4.5.0; R Development Core Team, 2020). For the Hungarian sequences, the best nucleotide substitution model was selected by the Maximum-Likelihood analysis implemented in MEGA12 using the Bayesian Information Criterion (BIC) as suggested [57]. Relationships between the haplotypes were also calculated and visualized by constructing a haplotype network using the median-joining method available in PopART version 1.7 [60], setting ε = 10 and transversions/transition weighting of 3:1.

To investigate the hierarchical distribution of genetic variance, an analysis of molecular variance (AMOVA) was computed using the poppr (version 2.9.8) package [61] in the R environment (version 4.5.0; R Development Core Team, 2020). The analysis was run for 10,000 permutations to evaluate the significance of each parameter. For a hierarchical analysis, the populations were grouped according to regions: TD-S and TD-N into Transdanubia, NHM-Bö, NHM-Cs, NHM-M and NHM-Bü into North Hungarian Mountains and GHP into Great Hungarian Plain.

## 3. Results

The detailed geographic assignments, the genetic admixture proportions (q^(i)^ values) derived from the nuclear microsatellite analysis, and the identified mitochondrial haplotypes and haplogroups of the field wildcat individuals investigated in this study are summarized in Supplementary Table S1.

### 3.1. Microsatellites

For both domestic cats and wildcats, all investigated loci were polymorphic. In the wildcat groups, the lowest per-locus allele count was three (locus FCA220 in the NHM-M group) and the highest was 14 (loci FCA223 and F115, both in the GHP group); averaged over all 14 loci, the mean number of alleles per group ranged from 7.07 to 10.79. The extremes occur at different loci—within the NHM-M group alone, FCA220 carried three alleles and FCA096 carried 13—so the per-locus and mean values are not contradictory. Mean observed heterozygosity (H_O_) was 0.73–0.82 (in the North Hungarian Mountains—Börzsöny and Transdanubian-South groups), while mean expected heterozygosity (H_E_) was 0.76–0.82 (in the North Hungarian Mountains—Bükk and Great Hungarian Plain groups). Observed heterozygosity (H_O_) was higher than expected heterozygosity (H_E_) only in Southern Transdanubia (TD-S) and the Bükk group (NHM-Bü), whereas H_E_ was higher than H_O_ across all remaining wildcat groups. The effective number of alleles ranged from 4.56 to 5.88 (Supplementary Table S3). Departures between H_O_ and H_E_ at individual loci were bidirectional—54% of the locus × group combinations showed H_O_ < H_E_ and 46% showed H_O_ > H_E_—and averaged over the 14 loci the two statistics were closely similar in every group, with a mean heterozygote deficit across the wildcat groups of 3.5%. A repeatable deficit was confined to a small number of loci, notably FCA220, consistent with the null alleles expected when markers developed for the domestic cat are applied to the wildcat; these were screened with MICRO-CHECKER and the population comparisons were repeated with the ENA correction implemented in FreeNA, which gave F*_ST_* values of the same magnitude as the uncorrected estimates. Treating spatially structured groups as single populations (a Wahlund effect, consistent with the significant differentiation among populations within regions, ΦSC = 0.083, *p* = 0.0226) will also contribute to the small positive values observed.

**Figure 2:**
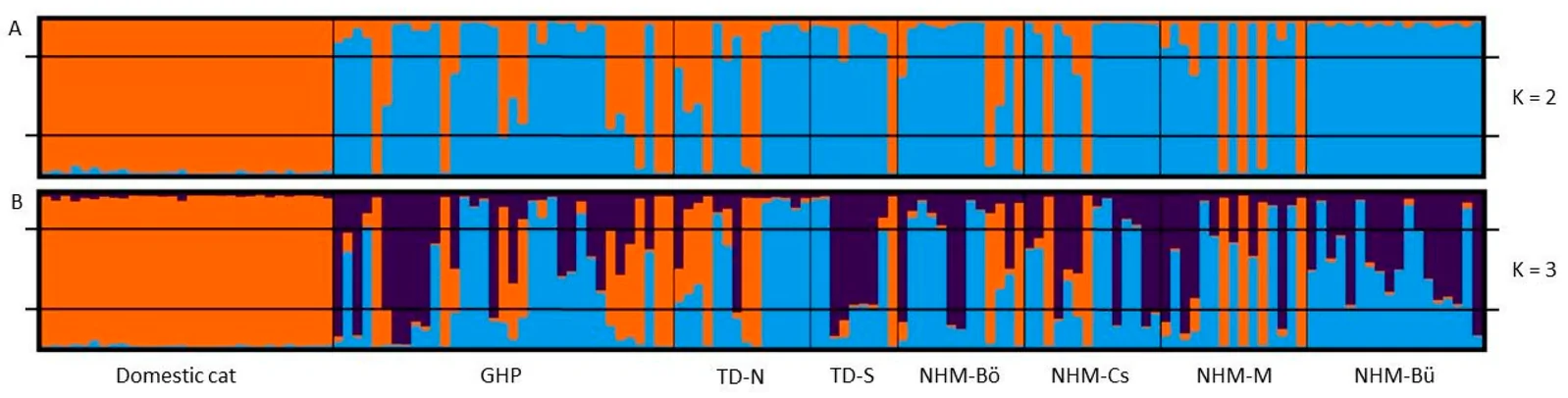
Admixture analyses of domestic and Hungarian free-living wildcats based on STRUCTURE outputs aligned and averaged across 10 independent runs per K using CLUMPAK for K = 2 (**A**) and 3 (**B**). Each individual is represented as a vertical bar partitioned into K coloured segments, with length proportional to the estimated membership fraction (q^(i)^) in each clusters. The horizontal black lines indicate values of individual proportion of membership q^(i)^ = 0.25 or 0.75. Orange indicates the domestic cat associated cluster and the blue the wildcat associated cluster at K = 2; at K = 3 the additional purple segment indicates the second cluster recovered within the field samples.

The STRUCTURE program showed the highest average log-likelihood values for two genetic clusters, and the second-order rate of change in the log-likelihood values was also highest for two genetic clusters, K = 2 (Figure 2A). In this case, domestic cat references clustered into one group, while the various wildcat groups clustered into another. Within the geographically defined wildcat groups, 18 individuals were genetically identified to the domestic cat cluster, and 16 individuals were identified as putatively admixed. The four supervised statistics of the Puechmaille method indicated the presence of three clusters across all thresholds, K = 3 (Figure 2B). In this case, domestic cats and wildcats formed distinct genetic clusters; however, the internal genetic structure observed within the wildcat group (K = 3) did not correlate with geographical location. Because these internal wildcat genetic lineages do not form geographically segregated populations across the study area, all subsequent population genetic analyses were carried out under the K = 2 model, assuming that wildcats represent a single polymorphic population. Within the geographically defined wildcat groups, domestic cat (*n* = 18) and putatively admixed individuals (*n* = 10) were also identified under this model (Figure 2B).

**Figure 3:**
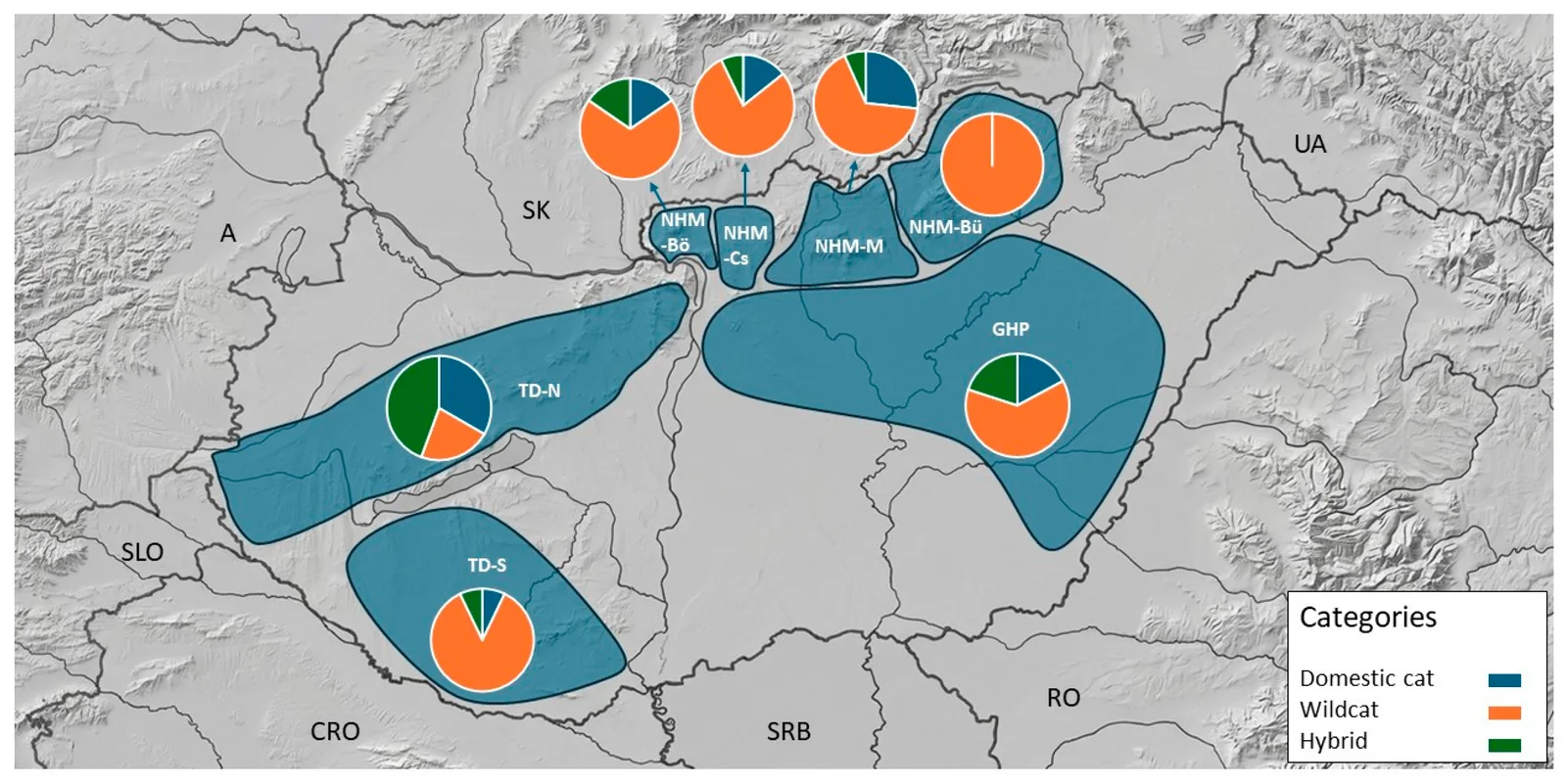
Distribution map of the assigned genetic categories across the sampled regions. Pie charts show the proportional frequency of domestic cats, wildcats, and putatively admixed individuals within each specified geographic group based on the Structure clustering analysis. Blue shaded areas indicate the approximate geographic boundaries of the sampled sub-regions (TD-N, TD-S, NHM-Bö, NHM-Cs, NHM-M, NHM-Bü, and GHP).

Based on the results of the Structure clustering analysis, the NHM-Bü sub-region exhibited the lowest number of putatively admixed and domestic cat individuals among the investigated geographic groups (*n* = 0 in both categories). In contrast, the highest number of putatively admixed individuals was identified in the GHP population (*n* = 7; 20%), which also presented the highest occurrence of domestic cats (*n* = 6; 17.14%). Examining the further areas, out of the 9 samples from the TD-N region, 2 (22.22%) were genetically assigned as wildcats, 4 (44.44%) as putatively admixed individuals, and 3 (33.33%) as domestic cats. The vast majority of the 14 animals originating from the TD-S region were wildcats (*n* = 12; 85.71%), alongside which only 1 putatively admixed individual (7.14%) and 1 domestic cat (7.14%) were identified. Among the 13 analyzed specimens from the NHM-Bö group, 9 (69.23%) were classified as wildcats, while 2 individuals each (15.38%) were assigned as putatively admixed individuals and domestic cats. Within the 14 samples from the NHM-Cs group, 11 (78.57%) resulted as wildcats, 1 as a putatively admixed individual (7.14%), and 2 as domestic cats (14.29%). Finally, out of the 15 individuals in the NHM-M region, 10 (66.67%) represented the wildcat lineage, while an additional 1 putatively admixed individual (6.67%) and 4 domestic cats (26.67%) were detected (Figure 3).

**Figure 4:**
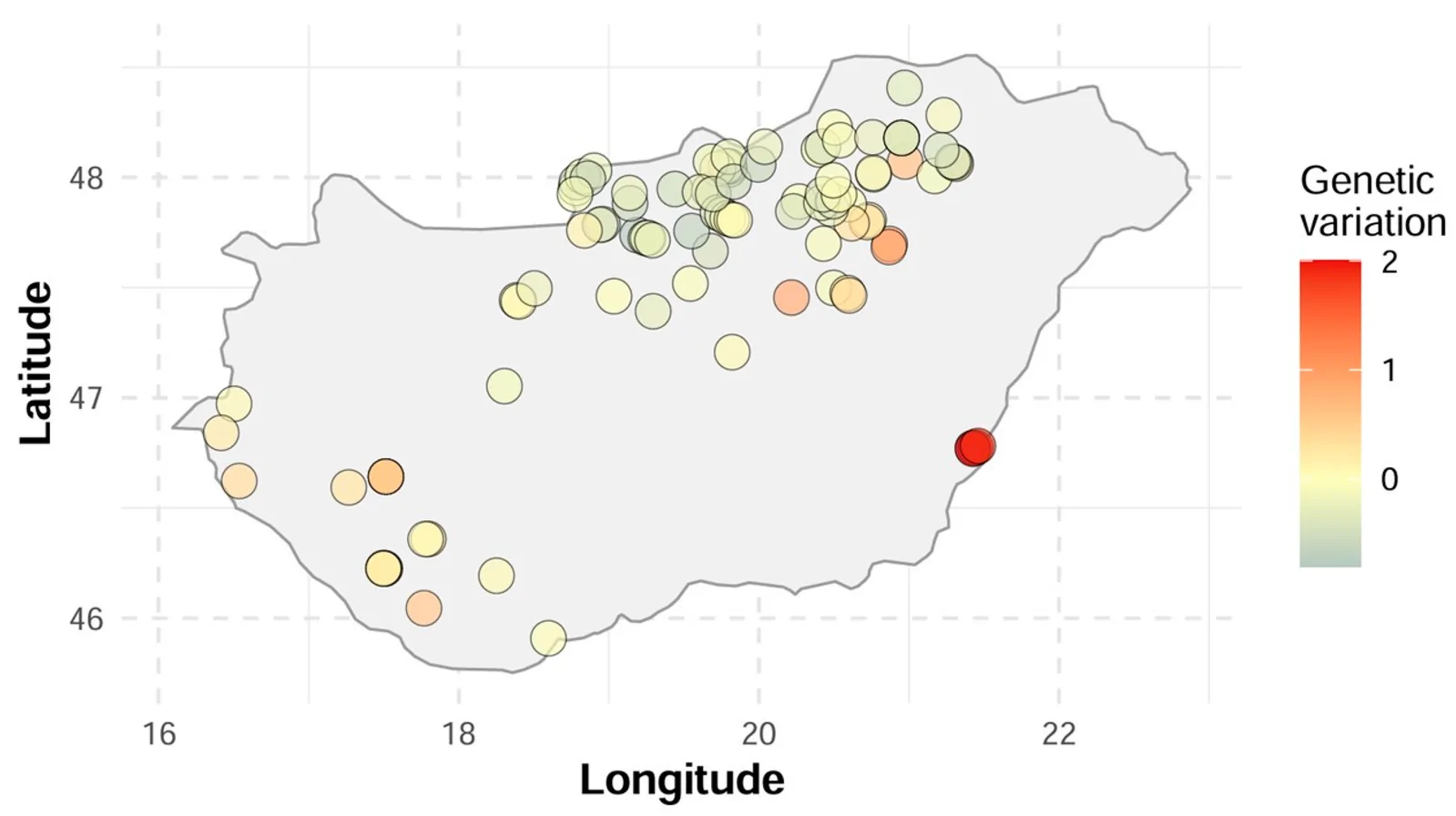
Spatial genetic structure of Hungarian wildcat populations based on spatial Principal Component Analysis (sPCA). The map illustrates the spatial distribution of genetic variation across Hungary. The colour scale represents the principal component scores calculated from the sPCA, where points with similar colour gradients indicate higher genetic similarity between geographically proximate individuals. A spatial pattern is discernible, with differentiation observed between samples originating from the northern, western, and southwestern regions of the country, reflecting the underlying spatial patterns of genetic variance.

Based on the results of the spatial Principal Component Analysis (sPCA), the genetic variability of the Hungarian wildcat population is not distributed randomly across space. A spatial genetic structure can be visually identified in Figure 4. Spatially close individuals (indicated by points with similar colour shades) exhibit higher genetic similarity, whereas distinct genetic differentiation is evident among populations located in more distant regions of the country. This visually identified spatial structure was statistically corroborated by the Mantel-test results. A significant positive correlation was detected between genetic and geographic distances (r = 0.118, *p* = 0.005). This association is statistically significant but weak, and is consistent with a pattern of isolation by distance (IBD) within the sampled population rather than demonstrating it conclusively.

Genetic distances among the investigated populations were estimated using pairwise *F*_ST_ values corrected for the presence of null alleles (FreeNA) (Table 1). The results revealed that the domestic cat population is strongly and highly significantly differentiated from all other investigated groups (*F*_ST_ = 0.034–0.139; *p* < 0.01 and *p* < 0.001). The highest degree of differentiation within the entire dataset was observed between the domestic cat and the NHM-Bü population (*F*_ST_ = 0.139, *p* < 0.001), most likely because no domestic cat or putatively admixed individuals were identified within this group. Genetic structuring among the non-domestic groups was more moderate but well-defined. The NHM-Bü population exhibited statistically significant, moderate differentiation from the other groups (*F*_ST_ = 0.026–0.062; *p* < 0.05–0.001). In contrast, among certain geographic or categorized population pairs—particularly among the NHM-Bö, NHM-Cs, and NHM-M groups—pairwise *F*_ST_ values were not significantly different from zero (*F*_ST_ ≤ 0.017; *p* > 0.05; negative estimates were treated as zero). No significant differentiation was therefore detected among these groups, which is consistent with gene flow between them; given the small and unequal group sizes, however, this result should not be read as positive evidence for the absence of barriers to gene flow.

The off-diagonal elements represent genetic distances between the specified population groups. Asterisks indicate the level of statistical significance: *** *p* < 0.001, ** *p* < 0.01, * *p* < 0.05, while “ns” represent non-significant differentiation (*p* > 0.05). GHP (Great Hungarian Plain), TD-N (Transdanubia North), TD-S (Transdanubia South), NHM-Bö (North Hungarian Mountains—Börzsöny), NHM-Cs (North Hungarian Mountains—Cserhát), NHM-M (North Hungarian Mountains—Mátra), and NHM-Bü (North Hungarian Mountains—Bükk).

Using the previously applied assignment probability of q^(i)^ ≥ 0.75, 100% of the generated wildcat genotypes assigned to one cluster, and 100% of the domestic cat genotypes assigned to the other cluster. Among the generated F1s and F2s, 100% and 90% of the genotypes, respectively, were identified as putatively admixed individuals; none of the F1 genotypes was assigned to a parental cluster, whereas the remaining 10% of the F2 genotypes were assigned to the domestic cat cluster. Of the generated second backcrosses, 76% and 84% assigned to the corresponding parental cluster, whereas 24% and 16% failed to assign. 100% of the third backcrosses assigned to the appropriate parental group, with no generated genotypes remaining unassigned. Detailed information regarding the Structure q^(i)^ values for the generated genotypes is provided in Supplementary Tables S4 and S5.

### 3.2. Phylogenetic Analysis

By analyzing the 669 bp sequence portion, we identified eight haplotypes among the 118 samples. The aligned sequences contained 16 polymorphic sites, corresponding to 16 mutations; under an infinite-sites model without recombination this number of segregating sites can support up to 17 distinct haplotypes, of which eight were observed here. The total haplotype diversity (Hd) of the analyzed dataset was 0.683 ± 0.036, and the nucleotide diversity (*π*) was 0.00704 ± 0.00062. The average number of nucleotide differences (*k*) among individuals was 4.712. Demographic statistics for the overall dataset did not show significant deviations from the mutation-drift equilibrium, yielding a Tajima’s D value of 1.5719 (*p* = 0.1160) and a Fu and Li’s F* value of 0.6815 (*p* = 0.4617) (Table 2).

The eight identified haplotypes belonged to four main haplogroups: sub-clade Ia (*n* = 60; 50.85%) was represented by the dominant haplotype dw4, followed by haplogroup III (*n* = 25; 21.19%, driven mainly by haplotype dw1, *n* = 22; 18.64%), haplogroup V (*n* = 15; 12.71%; w2), haplogroup IV (*n* = 12; 10.17%; w1), and sub-clade Ib (*n* = 6; 5.08%; dw3), while haplotype dw5 was found in only a single sample (0.85%). Two new haplotypes (Hap1 and Hap7) were identified, both belonging to haplogroup III and each represented by a single sample (0.85%).

**Figure 5:**
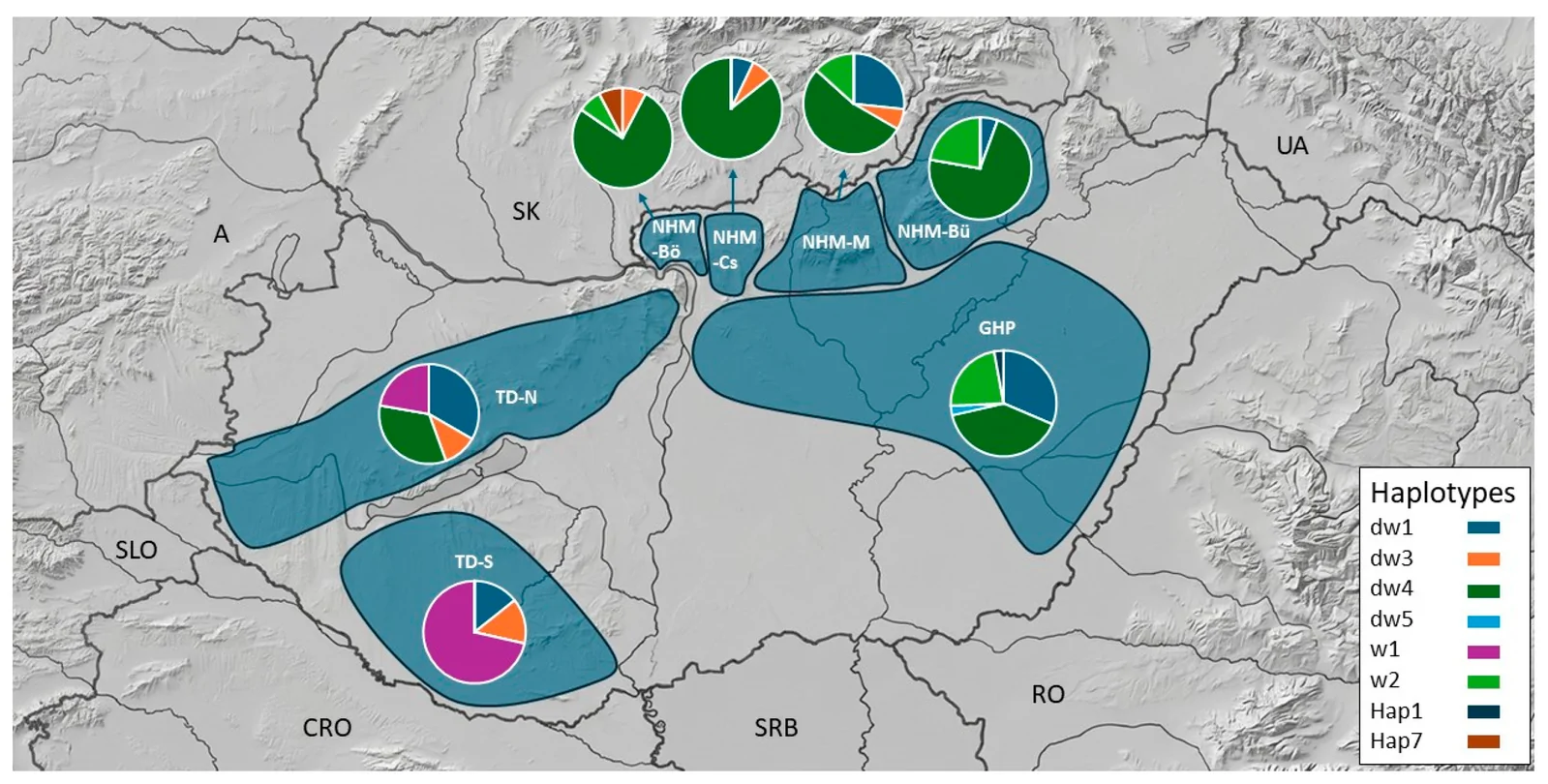
Distribution map of the identified haplotypes across the sampled regions. Pie charts show the proportional frequency of haplotypes within each designated geographic group. Blue shaded areas indicate the approximate geographic boundaries of the sampled sub-regions (GHP, TD-N, TD-S, NHM-Bö, NHM-Cs, NHM-M and NHM-Bü).

When the dataset was partitioned into the seven geographic sub-regions, the number of identified haplotypes ranged from three (TD-S, NHM-Cs, and NHM-Bü) to five (GHP) (Figure 5). Haplotype dw4 predominated across the NHM populations (NHM-Bö, NHM-Cs, NHM-M, and NHM-Bü), whereas dw1 was most common in the GHP group, and both dw1 and dw4 were equally prevalent in TD-N. In contrast, the TD-S population was characterized by a predominance of haplotype w1. Genetic variability was highest in the TD-N and GHP sub-regions, both in terms of haplotype and nucleotide diversity, while the lowest diversity values were observed in the NHM-Cs and NHM-Bö groups (Table 2). Demographic statistics (Tajima’s D and Fu and Li’s F*) did not reveal significant deviations from equilibrium in any sub-region (*p* > 0.05), although the NHM-Bö group showed a negative trend approaching significance (Tajima’s D = −1.7203, *p* = 0.0854; Fu and Li’s F* = −1.9014, *p* = 0.0699).

### 3.3. Phylogeographic Analysis of Hungarian Wildcat and Putative Admixed Individuals

**Figure 6:**
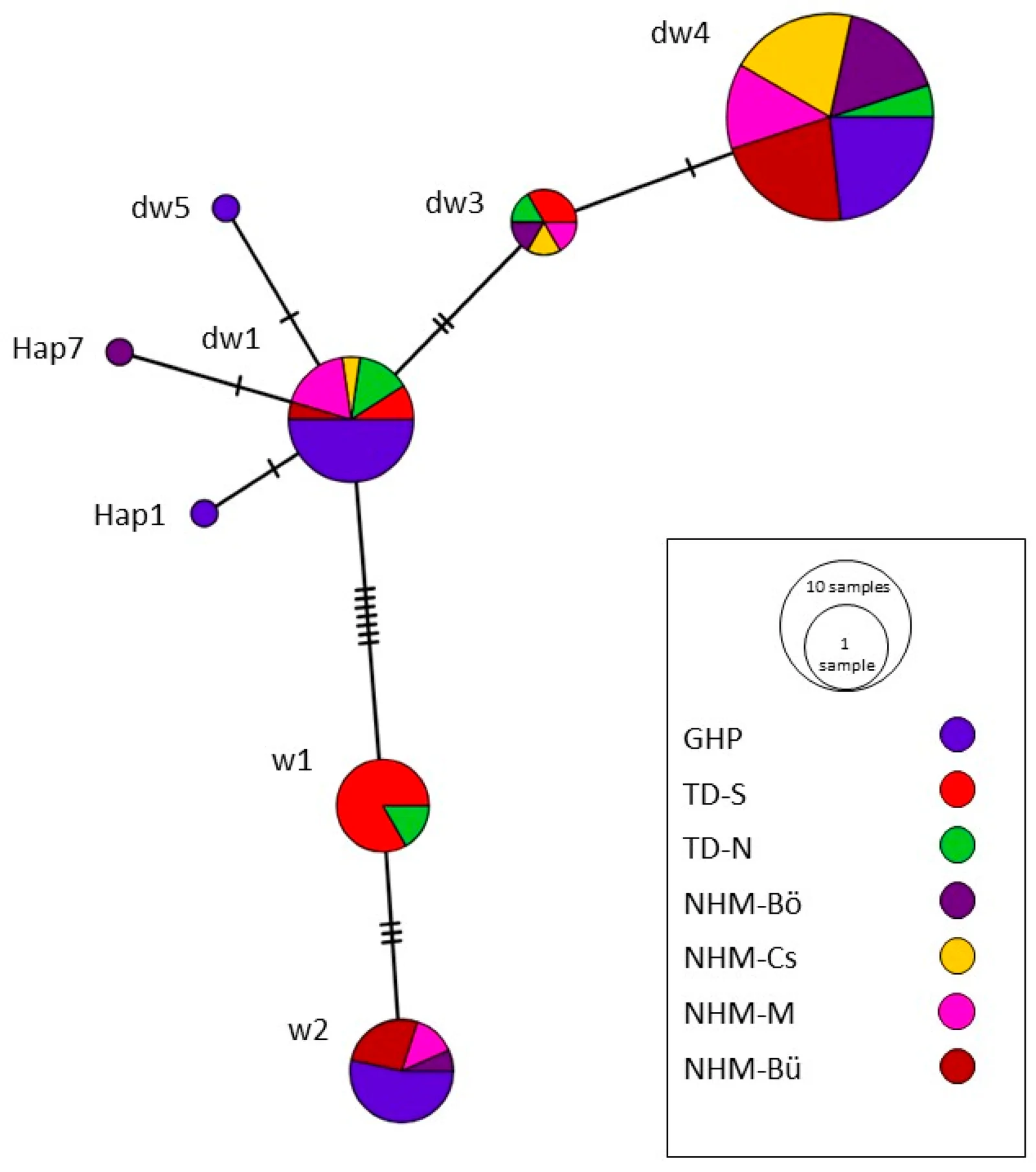
Median-joining (MJ) networks among the 8 mitochondrial haplotypes obtained from the Hungarian wildcat population. The frequency of each haplotype is proportional to the size of the circles. Each haplotype was partitioned and coloured according to the proportion of individuals belonging to each geographic sub-region (GHP: Great Hungarian Plain, TD-S: Southern Transdanubia, TD-N: Northern Transdanubia, NHM-Bö: North Hungarian Mountains Börzsöny, NHM-Cs: North Hungarian Mountains Cserhát, NHM-M: North Hungarian Mountains Mátra, NHM-Bü: North Hungarian Mountains Bükk). Small bars on the solid lines indicate the number of mutations between two different haplotypes.

The reconstructed MJ network, which includes the eight haplotypes (dw1; dw3; dw4; dw5; w1; w2; Hap1; Hap7) identified from the analyzed dataset, revealed a distinct genetic structure. The network topology is structured around two highly frequent and dominant haplotypes: dw4 and dw1. These two central variants are separated by a total of three mutational steps through an intermediate, lower-frequency haplotype (dw3), which is separated by one mutation from dw4 and two mutations from dw1. Node dw1 forms the centre of a star-like radiation: low-frequency, likely private or rare haplotypes (Hap1, Hap7, dw5) diverge from this central variant by a single mutational step. Conversely, w1 and w2 represent a genetically more distant, divergent branch. w1 is separated from the central dw1 variant by seven mutational steps, followed by three additional mutations leading to haplotype w2. The colour-coded geographic repartition highlights that the most frequent haplotypes (particularly dw4 and dw1) are not strictly isolated to a single geographic region but are shared among several sub-regions (e.g., the Great Hungarian Plain/GHP and various North Hungarian Mountains/NHM groups). This distribution pattern supports the presence of genetic connectivity and historical gene flow among the populations (Figure 6).

To investigate the hierarchical distribution of genetic variance, an analysis of molecular variance (AMOVA) was performed, revealing that the great majority of the variation was partitioned within samples (σ^2^ = 3.899; 77.88% of the total variance; Φ_ST_ = 0.221; *p* = 0.0001). Differentiation among populations within regions accounted for a small but statistically significant share of the variance (σ^2^ = 0.354; 7.08%; ΦSC = 0.083; *p* = 0.0226). In contrast, differentiation among the three geographic macro-regions explained 15.04% of the variance and was not statistically significant (σ^2^ = 0.753; ΦCT = 0.150; *p* = 0.1545) (Table 3).

## 4. Discussion

The microsatellite loci used in the genetic analysis of the Hungarian wildcat population—the majority of which were originally developed for the domestic cat [43]—performed adequately for estimating population genetic metrics. The average number of alleles (N_A_: 7.07–10.79) and the expected heterozygosity (H_E_: 0.76–0.82) measured in the Hungarian samples are high. This allelic diversity exceeds the values reported for several other European populations, such as those in Germany (H_E_: 0.65) and Italy (H_E_: 0.69) [62,63]. The Hungarian results are most comparable to values observed in France (N_A_: 7.0; H_E_: 0.73) and Croatia (N_A_: 7.2; H_E_: 0.72) [64,65]. It is crucial to note, however, that the seemingly higher diversity indices observed in wildcat samples collected from natural habitats often do not reflect population “health,” but rather suggest the presence of historical or recent introgression (gene flow) from domestic cats, as demonstrated by [33] in the case of Swiss populations.

Although STRUCTURE analysis (at K = 3) identified clusters within the free-living wildcat samples, these did not exhibit any distinct spatial patterns. These results suggest that genetic differentiation between certain regions (e.g., Börzsöny, Cserhát, Mátra) is limited, which suggests weak regional differentiation, substantial gene flow, and continuous admixture. The East European—and specifically the Hungarian—wildcat population is genetically highly admixed and integrated; thus, it cannot be differentiated into distinct biogeographic units, as has been documented in populations such as those in Scotland. This may result from long-standing hybridisation and introgression [4]. While clear genetic discontinuities caused by mountain ranges or major highways have been observed in other European populations [62,66], such significant natural or anthropogenic barriers to gene flow are absent among the study areas in Hungary.

In contrast, sPCA revealed high-resolution structure, as this method incorporates the geographic coordinates of the samples. Following the removal of identified domestic cat individuals, the Mantel-test suggest a significant but weak positive correlation between genetic and geographic distances (*r* = 0.118, *p* = 0.005). This suggests that isolation by distance is present. The Hungarian population is characterized not by strong genetic breaks, but by gradual, continuous transitions (clines), which may suggest well-connected habitats and high mobility of the species [66].

Based on these results, it can be stated that hybridisation between wildcats and domestic cats occurs in Hungarian natural populations. In our current study, we detected an intermediate assignment rate of 13.56% based on the STRUCTURE analysis, while free-ranging domestic cats identified in the field accounted for an additional 15.25% of the samples (bringing the combined non-wildcat proportion to 28.81%). As a methodological limitation, it should be noted that the parental wildcat reference individuals for the simulations were likewise selected based on a STRUCTURE q^(i)^ > 0.75 threshold rather than being independently validated pure wildcat references derived from genome-wide genomic methods. This is consistent with findings in various other European populations; however, the extent of hybridisation appears to vary significantly across regions. While hybridisation rates in Germany are relatively low, estimated at approximately 3% [27,32], higher levels indicating moderate hybridisation have been documented in other countries, such as Italy (3–11%), Switzerland (12.5–15%), and the Iberian Peninsula (14–21%) [11,18,32,37,62,67]. Refs. [64,65] reported hybridisation rates of approximately 29% in France, with figures ranging from 13% to 52% recorded across Balkan populations. The most extreme rates of hybridisation have been observed in Scotland, where values can reach 80–90% [12,24]. In Hungary, preliminary studies conducted in the mid-2000s estimated hybridisation rates between 25% and 31% using genetic methods [4,30].

During the 18th and 19th centuries, the European wildcat was subjected to intensive hunting, leading to drastic population declines, local extinction, and fragmentation [68]. When population densities reach critical levels, the scarcity of conspecifics increases the likelihood of mating with the ubiquitous domestic cat [4]. Deforestation and habitat fragmentation have forced wildcats into small, isolated habitat [68]. This contributes to a decline in genetic diversity and heightens the risk of hybridisation, particularly at habitat borders [30]. The mosaic of agricultural areas and forest patches (agro-forestry ecosystems) facilitates the interaction of the domestic cat and wildcat. While continuous forests provide refuge for wildcats, settlements and agricultural lands serve as centres for domestic cats and hybrids; within these contact zones, hybridisation becomes more frequent [17,24]. The extensive hybridisation observed in Hungarian wildcats was historically predestined by habitat loss and population declines resulting from persecution. This demographic crisis, compounded by the mosaic landscape structure characteristic of Hungary, enables increased contact between wild and domestic cats along forest edges. Together with our current genetic findings, these factors substantiate the ancient and persistent nature of introgression, which basically threatens the genetic integrity of the local population [4,30].

In addition to the loss of genetic purity, this ongoing interbreeding can have serious ecological consequences. As a mesocarnivore, the wildcat provides essential top-down control of small mammal populations within forest ecosystems [22]. Extensive hybridisation may alter phenotypic traits, hunting behaviour, and habitat use, potentially shifting the ecological niche of admixed individuals closer to that of feral cats, thereby disrupting their native ecosystem function [14]. Therefore, strict and targeted conservation management is urgently required. Since our spatial analyses (sPCA) revealed gradual clines rather than sharp genetic breaks, management strategies must focus on the agro-forestry contact zones. To mitigate further introgression, conservation strategies in various European regions often focus on establishing buffer zones around core forest habitats [6,32]. Within these contact zones, wildlife management practices frequently involve the regulation or removal of free-roaming and feral domestic cats [32]. However, since morphological identification has proven unreliable in highly admixed populations [31], such removal efforts in several countries are increasingly supported by rapid genetic screening to avoid the accidental culling of pure wildcats [33]. Furthermore, international conservation frameworks highlight that public awareness campaigns targeting rural cat owners regarding responsible pet ownership (e.g., sterilization and indoor housing) serve as essential components of comprehensive management plans aimed at reducing the constant influx of domestic genes into the wildcat gene pool [12,27].

The mitochondrial DNA analysis of the Hungarian wildcat population revealed the presence of eight distinct haplotypes, which form a structured median-joining network centred around two dominant variants, dw4 and dw1. Evaluated within the broader European phylogeographic context, these haplotypes are clearly split into two deeply divergent evolutionary lineages: Lineage D, which encompasses the domestic cat and African wildcat origins, and Lineage W, which represents wildcat-assigned ancestry [10,35,36]. The widespread distribution of shared (dw) haplotypes across various Hungarian sub-regions initially appears to confirm extensive, country-wide introgression of domestic cats. However, a detailed phylogenetic assessment reveals a much more complex evolutionary history. One of the most notable phylogeographic patterns in the Hungarian populations the overwhelming dominance of the dw4 haplotype in the area of the North Hungarian Mountains (NHM-Bö, NHM-Cs, NHM-M, and NHM-Bü). This variant belongs to sub-clade Ia of lineage D, which, according to evolutionary models, diverged approximately 37,700 years ago [35]. Since this divergence long pre-dates currently known archeological and genetic evidence for cat domestication [10,69,70], the high frequency of dw4 in northern habitats does not necessarily suggest recent introgression with modern domestic cats. Rather, one possible explanation is a legacy of late Pleistocene, historical lineage retention and ancient introgression, when European wildcats migrating toward the Levant and Anatolia interbred with local *Felis lybica* populations [36,71,72]. This ancient introgression may reflect the mito-nuclear discordance observed in the regions of the North Hungarian Mountains, where individuals appear genetically pure based on nuclear microsatellites yet carry a shared mitochondrial pattern [26]. In contrast, the dw1 haplotype, predominantly found in the Great Hungarian Plain (GHP), reveals an entirely different evolutionary story. Based on demographic simulations, haplotypes such as dw1 and dw3 differentiated recently, about 1000 years ago, and may suggest recent human-mediated introgression originating from *Felis catus* [35]. This recent introgression is in perfect concordance with the high proportion of admixed individuals detected by nuclear STR analysis in the Great Hungarian Plain, reflecting intense anthropogenic pressure and frequent interactions with domestic cats in highly fragmented agro-forestry mosaic landscapes [4,6]. The ancestral evolutionary heritage of the European wildcat (Lineage W) in the Hungarian population is primarily preserved by the w1 and w2 haplotypes. Notably, the w1 haplotype is particularly frequent in Southern Transdanubia (TD-S), a region that also exhibits a high proportion of wildcat-assigned individuals based on nuclear results. According to the continental phylogeographic framework, the w1 variant belongs to clade IV, corresponding to a South-Eastern European spatial cluster [35]. The dominance of this wildcat-assigned lineage in southern Hungary likely reflects a post-glacial recolonization wave originating from Balkan or extra-Mediterranean glacial refugia following Pleistocene climatic oscillations [4,29,73]. The complex yet continuous nature of these genetic patterns is further supported by the analysis of molecular variance (AMOVA). The hierarchical partitioning of genetic variance showed that the vast majority of variation (77.88%) is found within individual samples, whereas genetic differentiation among the main geographic regions (15.04%) was not statistically significant. The absence of sharp, large-scale regional isolation correlates with the topology of the median-joining network, which clearly illustrates that dominant haplotypes are heavily shared across geographically distant sub-regions. Overall, these results suggest that the Hungarian wildcat population has not been characterized by long-term geographic isolation; its genetic structure has been continuously shaped by the species’ high mobility, historical recolonization waves, ancient inter-taxon gene flow, and varying local intensities of recent anthropogenic hybridisation [35,66].

## 5. Conclusions

Our study provides a comprehensive overview of the genetic diversity, spatial structure, and ecological challenges posed by hybridisation in the Hungarian European wildcat population. The patterns compatible with historical lineage retention and ancient introgression observed in the North Hungarian Mountains (presumably originating from past admixture with the *Felis lybica* lineage) and the recent hybridisation with domestic cats detected in the Great Hungarian Plain well reflect the complex evolutionary and demographic history of the population. Spatial analyses (sPCA) did not reveal sharp genetic barriers among the sampled Hungarian habitats; the population is characterized by continuous spatial transitions and isolation by distance, consistent with spatial gene flow across the landscape. Nevertheless, extensive hybridisation severely threatens the genetic integrity of wildcats and their ecological role in regulating small mammal populations within forest ecosystems. Identification based on morphological traits is no longer reliable due to the high degree of admixture. Therefore, future conservation strategies must focus on establishing buffer zones in critical agro-forestry contact zones, supporting the regulation of feral domestic cats through rapid and reliable genetic screening methods, reconsidering the current legal regulation of naturally occurring putatively admixed individuals, and launching public awareness campaigns on responsible pet ownership (e.g., spaying/neutering, indoor housing), which are essential to halt the continuous influx of domestic cat genes. The combined analysis of microsatellite loci and the mitochondrial *ND5* gene suggests that the sampled population retains high genetic diversity at the markers examined, and is compatible with the presence of both historical and recent introgression. Given the sample size and the resolution of the marker panel, these patterns should be regarded as indicative rather than conclusive. In particular, diversity estimates for sub-regions with small sample size (e.g., TD-N, *n* = 9) are subject to substantial sampling error and should be interpreted with caution rather than used as the basis for direct regional comparisons.

## Figures and Tables

**Table 1:** Pairwise *F_ST_* values calculated based on FreeNA correction for null alleles.

	Domestic Cat	GHP	TD-N	TD-S	NHM-Bö	NHM-Cs	NHM-M	NHM-Bü	
Domestic cat	0								Domestic cat
GHP	0.065 ***	0							GHP
TD-N	0.034 **	0.018 *	0						TD-N
TD-S	0.092 ***	0.023 **	0.021 *	0					TD-S
NHM-Bö	0.074 ***	0.007 ns	0.007 ns	0.022 **	0				NHM-Bö
NHM-Cs	0.093 ***	0.017 **	0.027 **	0.023 **	0.002 ns	0			NHM-Cs
NHM-M	0.072 ***	0.006 ns	0.017 ns	0.036 ***	−0.003 ns	−0.001 ns	0		NHM-M
NHM-Bü	0.139 ***	0.030 ***	0.062 ***	0.049 ***	0.026 **	0.032 ***	0.028 ***	0	NHM-Bü
	Domestic cat	GHP	TD-N	TD-S	NHM-Bö	NHM-Cs	NHM-M	NHM-Bü	

Key: ns = not significant, * *p* < 0.05, ** *p* < 0.01, *** *p* < 0.001.

**Table 2:** Summary of genetic diversity and demographic statistics across the seven geographic sub-regions and the overall dataset based on the analyzed 669 bp mitochondrial sequence.

Population	N	Number of Haplotypes	Haplotype Diversity (Hd ± SD)	Nucleotide Diversity (π ± SD)	Tajima’s D	*p*	Fu and Li’s F*	*p*
GHP	35	5	0.708 ± 0.037	0.00781 ± 0.00111	1.4215	0.1552	1.0409	0.1219
TD-S	14	3	0.484 ± 0.142	0.00539 ± 0.00146	1.05	0.2937	1.3591	0.0986
TD-N	9	4	0.806 ± 0.089	0.00648 ± 0.00169	0.8274	0.408	1.3439	0.249
NHM-Bö	13	4	0.423 ± 0.164	0.00395 ± 0.00234	−1.7203	0.0854	−1.9014	0.0699
NHM-Cs	14	3	0.275 ± 0.148	0.00082 ± 0.00051	−1.2783	0.2012	−1.1412	0.1195
NHM-M	15	4	0.667 ± 0.099	0.00604 ± 0.00206	0.0394	0.9686	1.1363	0.6969
NHM-Bü	18	3	0.451 ± 0.117	0.00738 ± 0.00207	1.1399	0.2543	1.4633	0.0954
Overall	118	8	0.683 ± 0.036	0.00704 ± 0.00062	1.5719	0.116	0.6815	0.4617

N: number of samples; Hd: haplotype diversity; π: nucleotide diversity; SD: standard deviation; p: significance level (*p*-value). Tajima’s D and Fu and Li’s F* values were calculated to test for mutation-drift equilibrium and potential population size changes.

**Table 3:** Results of the hierarchical analysis of molecular variance (AMOVA) assessing the genetic structure of the investigated regions and populations.

Source of Variation	df	SS	MS	σ^2^	%	Φ	*p*
Between regions	2	77.29	38.645	0.753	15.04	ΦCT = 0.150	0.1545
Between populations	4	35.336	8.834	0.354	7.08	ΦSC = 0.083	0.0226
Within samples	111	432.764	3.899	3.899	77.88	ΦST = 0.221	0.0001
Total	117	545.39	4.661	5.006	100		

df = degrees of freedom, SS = sum of squares, MS = mean square, σ^2^ = variance component, % = percentage of total variance, Φ = fixation index (ΦCT among regions, ΦSC among populations within regions, Φ_ST_ within samples), *p* = *p*-value.

## Data Availability

The eight identified mitochondrial *ND5* haplotypes presented in this study have been deposited in the NCBI GenBank database under the accession numbers (PZ832364-PZ832365, QB043413-QB043418).

## Supplementary Materials

The following supporting information can be downloaded at https://www.mdpi.com/article/10.3390/ani16203166/s1, Table S1: Detailed geographic and genetic information of the analyzed field wildcat samples; Table S2: Sequences of the primer pairs adapted and optimized from [43]; Table S3: Polymorphism of 14 autosomal microsatellite loci in domestic cat group and wildcat groups; Table S4: Genotypes generated using recom-sim.py and assigned with Structure; Table S5: Mean q^(i)^-values for STRUCTURE and ranges of simulated genotypes for their respective hybrid category are shown.
